# Development of a Microforce Sensor and Its Array Platform for Robotic Cell Microinjection Force Measurement

**DOI:** 10.3390/s16040483

**Published:** 2016-04-06

**Authors:** Yu Xie, Yunlei Zhou, Yuzi Lin, Lingyun Wang, Wenming Xi

**Affiliations:** Department of Mechanical and Electrical Engineering, Xiamen University, Xiamen 361005, China; 19920141152932@stu.xmu.edu.cn (Y.Z.); Linyz@xmu.edu.cn (Y.L.); wangly@xmu.edu.cn (L.W.); wmxi@xmu.edu.cn (W.X.)

**Keywords:** cell-holding device, cellular force sensor, force measurement, microinjection, micromanipulation, PVDF film

## Abstract

Robot-assisted cell microinjection, which is precise and can enable a high throughput, is attracting interest from researchers. Conventional probe-type cell microforce sensors have some real-time injection force measurement limitations, which prevent their integration in a cell microinjection robot. In this paper, a novel supported-beam based cell micro-force sensor with a piezoelectric polyvinylidine fluoride film used as the sensing element is described, which was designed to solve the real-time force-sensing problem during a robotic microinjection manipulation, and theoretical mechanical and electrical models of the sensor function are derived. Furthermore, an array based cell-holding device with a trapezoidal microstructure is micro-fabricated, which serves to improve the force sensing speed and cell manipulation rates. Tests confirmed that the sensor showed good repeatability and a linearity of 1.82%. Finally, robot-assisted zebrafish embryo microinjection experiments were conducted. These results demonstrated the effectiveness of the sensor working with the robotic cell manipulation system. Moreover, the sensing structure, theoretical model, and fabrication method established in this study are not scale dependent. Smaller cells, e.g., mouse oocytes, could also be manipulated with this approach.

## 1. Introduction

With the fast development of cell biology and related biotechnology, robot-assisted cell manipulation, which is precise and can enable a high throughput, is attracting interest from researchers [1,2,3,4]. Robotic cell manipulation, e.g., cell probing, grasping, or microinjection, requires an end effector to establish soft physical contact with the cell. A cell force sensor is, therefore, a crucial element, as it allows the robot to better understand and control the mechanical interaction between cell and manipulator.

**Figure 1:**
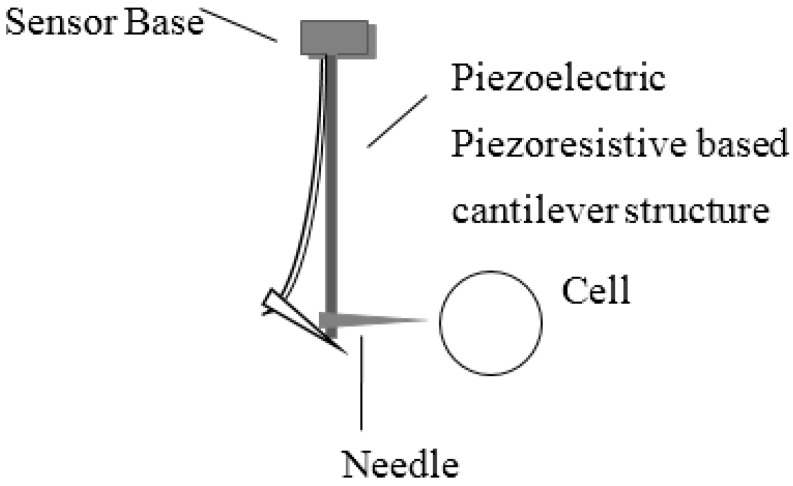
Typical probe based micro-force sensor.

Many force sensors have been developed for single cell force measurement. These include atomic force microscopes, optical tweezers and magnetic tweezers, which provide small force measurement at or below the nanonewton range. For larger cellular force measurement (in the micronewton range), a set of mechanical sensors has been developed using microelectromechanical systems (MEMS) fabrication techniques. A probe with a flexible beam is used as the force load of the cell; the force between the probe and the cell results in deformation of the beam, as shown in Figure 1. A force-displacement sensing mechanism, e.g., a piezoelectric [2,3,4,5,6], strain gauge [7,8,9], or capacitive transducer [10] is employed and microfabricated as the beam. Lu *et al.* [7] developed a piezoresistive micro-force sensor to monitor zebrafish embryo injection forces. A micropipette was bonded to the free end of a sensing beam, while the whole sensor was mounted on a movable stage. When the micropipette contacted the cell, the beam was deformed, and the change in resistance was measured using a Wheatstone bridge circuit. Rajagopalan *et al.* [9] used a flexible beam attached to a rigid probe to study the mechanical response of embryonic *Drosophila* axons. However, conventional micro-force sensors share some common limitations, which prevent their integration in a cell microinjection robot. In cell microinjection, the probing needle must be connected to the pressure tube so that a liquid substance can be injected into the cell (see also Figure 1). Since the needle is connected to a flexible beam, the gravity of the pressure tube is far greater than the injection force, *i.e.*, of the order of micro-newtons. In a study by Liu *et al.* [11], a vision-based force measurement method was developed by positioning the force-sensing structure at the cell side instead of the needle beam. An elastic polydimethylsiloxane (PDMS) pillar array was placed at the cell side and the slender forces of these pillars were measured during cell microinjection. The method overcomes the tube gravity problem associated with the conventional cell force sensor and can be used in a robot-assisted cell microinjection system, but compared with MEMS-based force sensing, it is difficult to precisely calculate the contribution of the pillar deflection by a visual tracking algorithm [12]. In this paper, we present a new design of a MEMS-based micro-force sensor capable of providing real-time cell force feedback in cell microinjection manipulation. A novel supported beam is designed for the cell holder side. The piezoelectric material polyvinylidine fluoride (PVDF) is chosen as the sensing element. Compared with vision-based elastic deformation sensing [11], PVDF-based force measuring offers a more rapid measurement response.

In addition, in the study of cell mechanics it has been found that the activity of cells, such as cell growth, division, signal conversion and gene expression, changes the cell’s mechanical properties, and *vice versa* [5]. The stiffness (ratio of stress to strain) of a developing egg changes at various stages of the cell cycle. For a zebrafish embryo, for example, the stiffness at the blastula stage is 1.66 times that at the prehatching stage [6]. From the robotic point of view, the manipulated object (*i.e.*, the cell) is time-varying and individually variable. Understanding the cell mechanical characteristics via a force sensor is thus a key step for the controller design in a robot manipulation system. To this end, a trapezoidal array based cell-holding device is specially designed. Combined with the micro-force sensor, this will improve the measurement speed and allow the researchers better understand the single cell characteristics by comparing the force characteristics of a batch of cells at same robotic manipulation environment. We demonstrate the suitability of these sensors in a robot-assisted cell microinjection application by determining the real-time injection force on zebrafish eggs.

## 2. Sensor Design

### 2.1. Sensor Construction

**Figure 2:**
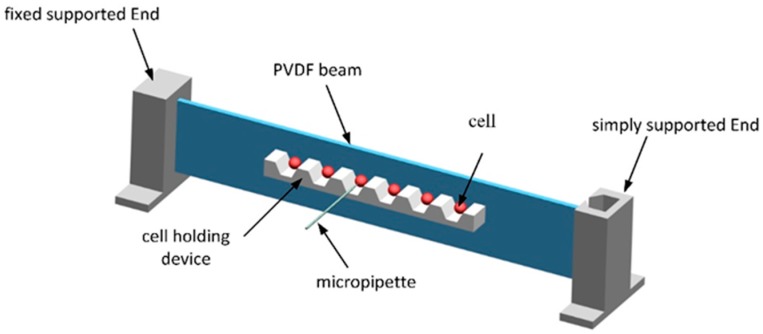
Sensor design.

In the cell microinjection process, a high-sensitivity sensor is required, as the cell load force is usually of the order of micronewtons or less. Figure 2 illustrates the structure of the proposed microforce sensor, which is composed of three parts: a mechanical transducer, which converts the needle injection force to deformation of the PVDF beam; an electrical transducer, which converts the charge output of the PVDF film to a corresponding voltage signal; and a cell-holding device. The principle of operation of the force sensor is as follows. When the needle penetrates the egg, the process can be considered quasi-static. The egg is suspended in culture medium, so the friction forces between the cell and the cell-holding device and the fluid dynamic forces can be ignored. According to Newton’s law, the needle probing force equals the force applied on the beam. The piezoelectric PVDF film is a lightweight and compliant film that directly attaches to the beam without disturbing the mechanical motion. As a typical piezoelectric material, it has a wide frequency bandwidth from 0.001 to 10^9^ Hz and a high voltage output. Thus, we designed a PVDF beam structure that is fixed at one end and simply supported at the other end. A U-shape constraint is designed at the non-fixed end of the beam to prevent unwanted disturbances.

In our design, the cell-holding device is firmly glued to the PVDF beam so that the center of the cell lies on the latitudinal center axis of the beam. When it is subjected to an injection force, the beam deforms. Since the micropipette is acting at the center of the cell, bending is the primary mode of deformation, while torsion is zero. Note that the sensitivity of the sensor output is determined by the width and length of the beam, the stiffness of the beam and the piezoelectric strain constant of the piezoelectric film. The structure of the beam is an important factor to be considered during design. Compared with a conventional cantilever beam structure, this beam design—fixed at one end and simply supported at the other end—is novel. Considering the mechanical properties of the materials and structural principles, the cantilever structure undergoes greater deformation and produces a higher voltage output than this novel beam design, for the same injection force. However, the asymmetrical nature of the bending of the cantilever produces a larger puncture wound to the cell than is produced by the novel beam design.

### 2.2. Microfabrication of Cell-Holding Device

**Figure 3:**
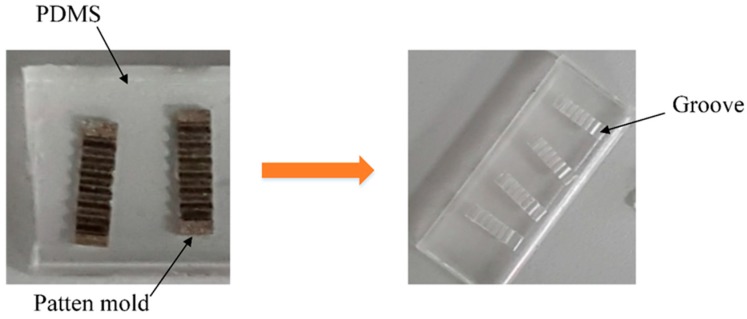
Fabrication of PDMS cell-holding device with stainless steel mold.

The cell-holding device was fabricated in two steps. First, an inverted trapezoidal grooved stainless steel mold was machined three times by electrical discharge machining with a cutting speed of 0.05 m/s. To ensure the transparency of the cell-holding device, electrolytic polishing was carried out, to reduce the surface roughness of the mold. The mold was then placed in the center of a glass Petri dish that had been cleaned with acetone. The second step was the preparation of the PDMS cell-holding device. Sylgard 184 pre-polymer and curing agent were fully mixed in a ratio of 10:1 by weight, and the mixture placed in a desiccator to degas for 20 min by allowing bubbles to escape. This mixture was then poured into a Petri dish and degassed in a vacuum heating furnace at 70 °C for 12 h. Using a sharp scalpel to cut around the pattern evenly and gently, the PDMS cell-holding device was removed from the stainless steel mold. Figure 3 shows the fabrication of the cell-holding device using the stainless steel mold.

### 2.3. Sensor Mechanical Model

**Figure 4:**
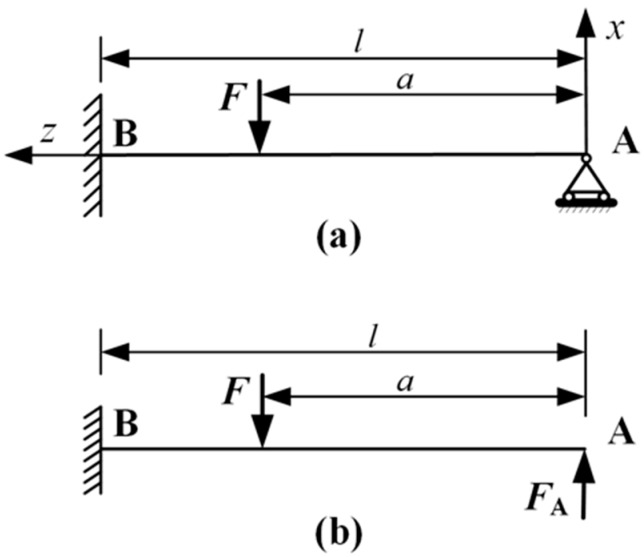
Simplified sensor structure (**a**) force at simple support; (**b**) force at clamped end. *F*, applied force; *F_A_*, force at *A* for zero movement at *A*; *a*, distance between forces; *l*, length of beam.

Since the PVDF force-sensing structure is newly designed for cell injection force measurement, we will analyze the force-mechanical model of the novel beam structure. The left end of the sensor in Figure 4 is the clamped end; the right end is the simply supported end.

For clear illustration, the beam of Figure 2 is redrawn in Figure 4a. A beam of length *l* and width *b* is considered. According to material mechanics theory of hyperstatic beams [13], the force *A* can be replaced by the force *F_A_* with the condition that the movement at point *A* is zero. Now the beam structure is simplified as a cantilever structure, as shown in Figure 4b. If the cantilever movement at point *A* is *W_A_*, then:

(1) $$W_{A}=W_{F}+W_{F_{A}}=0$$

where *W_F_* and *W_FA_* are the cantilever displacement at point *A* under the force *F* and *F_A_*, respectively. Then we get:

$$F_{A}=\frac{a^{2}(3l-a)}{2l^{3}}F$$

and the bending moment along the *z*-axis is:

(2) $$M(z)=\left\{ \begin{array}{l} \frac{Fza^{2}(3l-a)}{2l^{3}},0<z<a\\ Fa-\frac{Fz(2l^{3}+a^{3}-3la^{2})}{2l^{3}},a<z<l \end{array}\right.$$

where *a* is the distance from *F* to the supported end of the beam.

**Figure 5:**
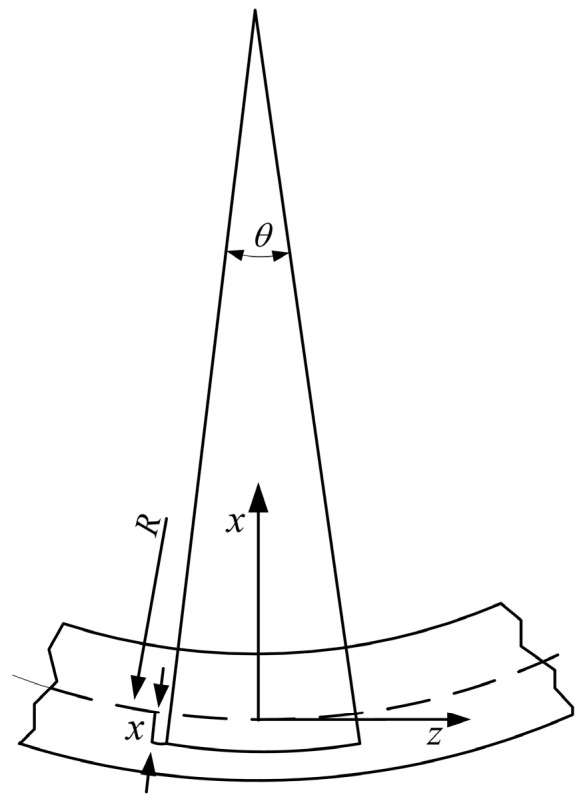
Beam stretching.

The bending moment causes tension at the bottom and compression at the top, as illustrated in Figure 5. The strain in a small element at a distance *x* from the neutral axis of the bent beam is:

(3) $$\varepsilon_{z}=\frac{(R+x)\theta -R\theta }{R\theta }=\frac{x}{R}$$

where *R* is the radius of the curvature of the neutral axis, and *X* and θ are defined in Figure 5.

The moment is expressed as:

(4) $$M(z)=\int_{s}x\sigma_{z}dxdy$$

where $\sigma_{z}$ is the stress in the *z*-direction.

According to Hook’s law and Equation (2), the stress in the *z*-direction is $\sigma_{z}=E_{b}\varepsilon_{z}$, where $E_{b}$ is the Young modulus of the beam. According to Equations (2)–(4), we have:

(5) $$R=-\frac{E_{b}I_{b}}{M(z)}$$

where *I_b_* is the inertial moment of the cross-sectional area. We then obtain a new expression for the strain:

(6) $$\varepsilon_{z}=\frac{xM(z)}{E_{b}I_{b}}$$

The force leading to the deformation of the material is mainly determined by the beam since the stiffness of the beam is much greater than that of the PVDF film. The strain of the PVDF film is dominated by the strain of the beam. Then the PVDF stress is $\sigma_{z-PVDF}=E_{PVDF}\varepsilon_{z}$, where $E_{PVDF}$ is the Young modulus of the PVDF film. Based on these equations, the stress of the PVDF film along the *z*-direction is given by:

(7) $$\sigma_{z-PVDF}=\left\{ \begin{array}{ll} \frac{FE_{pvdf}xza^{2}(3l-a)}{2E_{beam}I_{beam}l^{3}} & 0<z<a\\ \frac{E_{pvdf}x}{E_{beam}I_{beam}}[Fa-\frac{Fz(2l^{3}+a^{3}-3la^{2})}{2l^{3}}] & \;a<z<l \end{array}\right.$$

In our sensor construction, the PVDF beam is stretching along its length, so the charge *Q* is mainly affected by the piezoelectric strain constant *d*_31_. The electrical charge developed is:

(8) Q=∫sd31σz−PVDFdAQ=∫0b∫0aFEpvdfxzd31a2(3l-a)2EbeamIl3dzdy+∫0b∫alEpvdfxd31EbeamI[Fa-Fz(2l3+a3-3la2)2l3]dzdyQ=xEpvdfbd31(4Fal4-Fa3l2+Fa2l3-2Fl5)4EbeamIl3

Let *a* = *nl* (0 < *n* < *l*); then:

(9) $$Q=\frac{xbd_{31}l^{2}(4n-n^{3}+n^{2}-2)E_{PVDF}}{4I_{beam}E_{beam}}F$$

Since *d*_31_, *E_PVDF_*, *E_b_*, *n*, *x*, *l* and *b* are known constants, the relationship between the charge *Q* and *F* can be expressed as:

(10) $$Q=k_{1}F$$

where *k*_1_ is a constant.

### 2.4. Sensor Electrical Model

**Figure 6:**
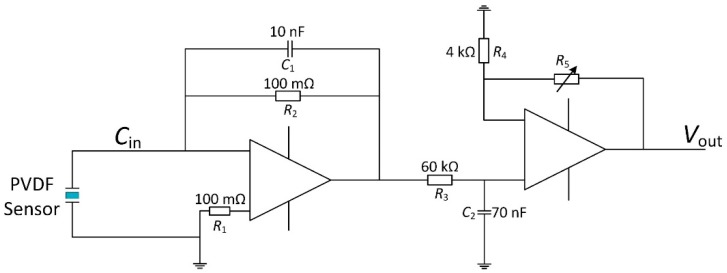
Electrical circuit of the sensor.

A PVDF film has a high direct current output impedance and appears as a charge source. Thus, a charge amplifier was added to convert the charge output from the PVDF film to a voltage signal. Following the charge amplifier, low-pass amplifiers were added to remove the power frequency interference in the circuit and amplify the weak voltage signal. In addition to the low-pass filters, shielding techniques were considered to prevent electromagnetic interference noise. The electrical circuit is shown in Figure 6.

The circuit operates by passing an input current that charges the feedback capacitor *C*_1_. Because of the virtual ground condition of the operational amplifier, the impedance of the parallel feedback resistor *R*_2_ and capacitor *C*_1_ is:

(11) $$Z=\frac{R_{2}}{1+j\omega R_{2}C_{1}}$$

By using Laplace transformation, the output voltage of charge amplifier *V*_1_ subjected to the input current d*Q*/d*t* is obtained as:

(12) $$V_{1}=\frac{R_{2}s}{1+R_{2}C_{1}s}Q$$

The charge amplifier network works as a high-pass filter where the cut-off frequency is:

$$f_{c}=\frac{1}{2\pi R_{2}C_{1}}=0.16Hz$$

It is worth noting that the input force is a limited-width signal in the time domain for force measurement. This means that the spectrum of the input signal is continuous and that some of the signal frequencies must be lower than the cut-off frequency. A digital compensator (1 + *s*)/*s* is thereby added to Equation (11) to let the sensor output *V*_1_ be proportional to charge *Q*. Then we have *V*_1_ = *k*_2_*Q*, where *k*_2_ = *R*_2_.

After there, a low-pass amplifier is then designed with an adjustable amplifier:

$$V_{out}=(1+\frac{R_{5}}{R_{4}})V_{1}$$

and a cut-off frequency:

$$f_{2}=\frac{1}{2\pi R_{3}C_{2}}=40Hz$$

to eliminate the power frequency interference. Combined with the charge amplifier, the transfer function of the complete electrical circuit becomes:

(13) $$V_{out}=k_{3}Q$$

where *k*_3_ is the electrical circuit gain. Substituting Equation (9) into Equation (12), the linear theoretical model for the PVDF force sensor is obtained as *V*_out_ = *k*_1_*k*_3_*F*, where the proportional parameter *k*_1_*k*_3_ can be obtained in calibration experiments.

### 2.5. Analysis of Force Sensor Configuration

In the sections above, we have obtained an analytical model of the injection force and the sensor voltage output of the PVDF beam structure. Because the V-shaped PDMS cell-holding device is attached to the beam, the beam bending caused by the force not only depends on the properties of the beam, but is also affected by the PDMS. Further analysis of the effect of the groove is obtained using finite element software ANSYS.

**Figure 7:**
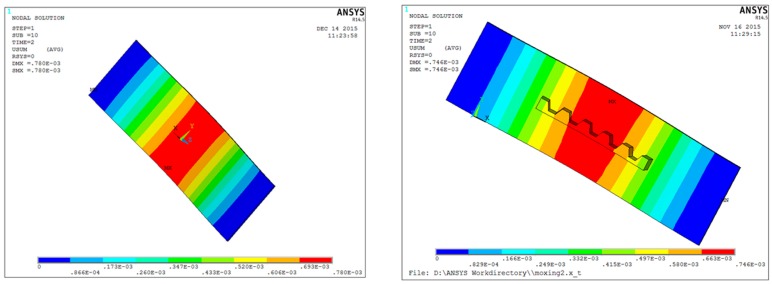
Strain distribution of the beam structure with (**left**); and without (**right**) cell-holding device.

The PVDF force beam structure models with and without PDMS were established, respectively, using finite element analysis. The suitable boundary conditions were defined using the software. The material is assumed to be isotropic and the analysis is of the linear type. The modulus of elasticity of the PDMS is 1.8 MPa, the density is 0.95 g/cm^3^, and the Poisson ratio is 0.48. The piezoelectric beam packaging material is polyethylene; its elastic modulus is 2200 MPa, its density is 1.01 g/cm^3^, and its Poisson ratio is 0.38. The material parameters are affected by the synthesis process and are not exact values. This does not affect the analysis of the influence of the groove on the bending of the beam, as they are generally sufficiently accurate under an external force of 0.2 N applied perpendicular to the same position of the beam (the center of the middle groove). Figure 7 shows the strain distribution of the PVDF beam structure with and without the PDMS cell-holding device. It can be seen that the forms of the bending is similar. The maximum deformation of the beam without the groove is 0.915 mm while that with the groove is 0.746 mm. This is mainly because the elastic modulus of the PDMS material is very small and far less than the modulus of elasticity of the packaging material. The packaging material has a large elastic modulus and is more rigid; the bending of the beam is mainly influenced by the more rigid material.

## 3. Calibration Experiments

**Figure 8:**
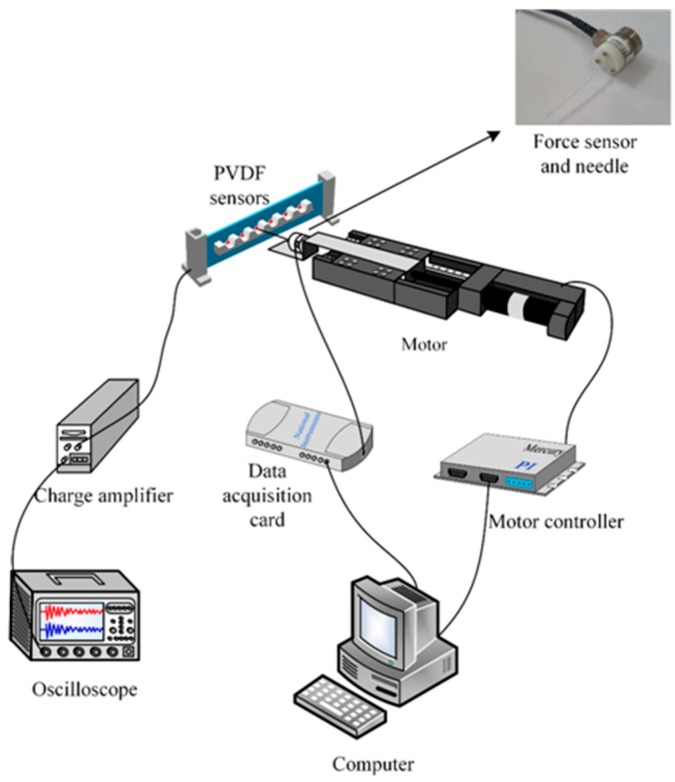
Schematic representation of the calibration set-up.

Prior to force-sensing application experiments, force calibration was performed to quantify the relationship between the applied force and sensor output. The calibration set-up is shown in Figure 8. When a needle moves towards the PVDF beam, the force exerted on the film equals that on the electrical scale. The needle was manipulated by a linear motor (M-403.4DG, PI, Karlsruhe, Germany) at a fixed speed, to ensure repeatability and consistency. A high-precision microforce sensor (Nano17, ATI, Apex, NC, USA) was attached to the needle to measure the needle force. An oscilloscope (DS1102E, Rigol, Beijing, China) was used to display the voltage signal of the electrical circuit. The PVDF film (SDT1-028K, MEAS., Hampton, VA, USA) has a length of 30 mm, a width of 13 mm and a thickness of 28 µm. The electrodes of the PVDF film were sealed with seal glue (HMG-628X, HOMEEN, Shengzheng, China), since the sensor is used in aqueous solution.

**Figure 9:**
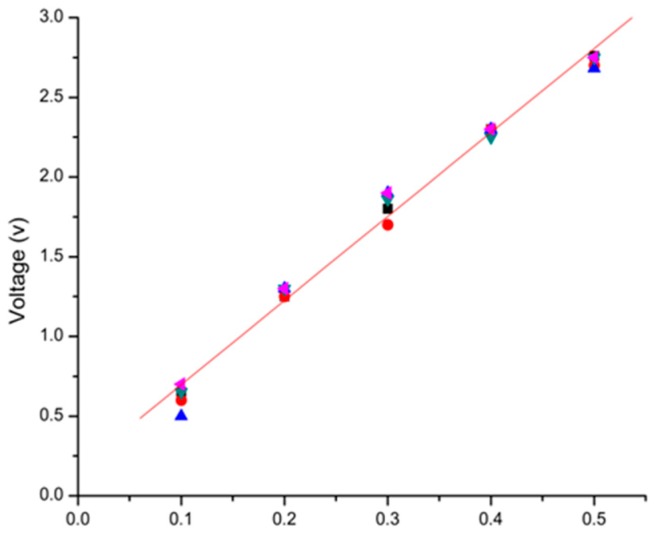
Displacement–voltage response of the sensor.

The relationship between needle movement and voltage is first examined. The needle was moved forward through different distances at a speed of 2.5 mm/s for five times. The five distances are 0.1 mm, 0.2 mm, 0.3 mm, 0.4 mm, and 0.5 mm, respectively. The corresponding output voltages are shown in Figure 9, which shows that the sensor output has high repeatability and linearity. Within the 0–0.5 mm measurement range, the linear sensitivity is 4.5 V/mm. The linearity is 1.82% full scale.

**Figure 10:**
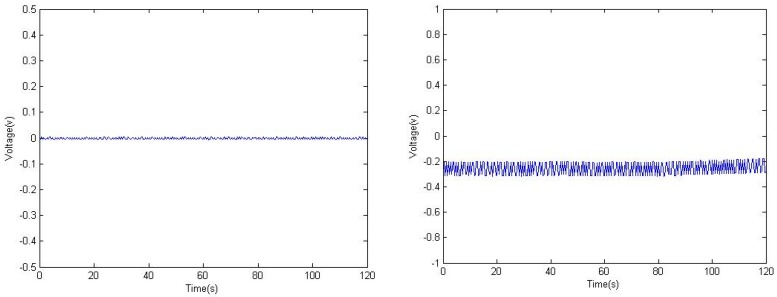
Voltage output at point 1 (**left**); and point V_1_ (**right**).

It is noticed that an offset exists in Figure 9. It may be from the capacitors since the PVDF, cable and charge amplifier contain ones. We observed the voltage output of the circuit at point 1 and *V*_1_ simultaneously without any force acts on the PVDF film in Figure 10. It can be seen that the offset comes from the PVDF and cable capacitors are almost undetectable while the offset at *V*_1_ is steady. This shows the offset may come from the non-ideal behaviour of the capacitor of *C*_1_.

**Figure 11:**
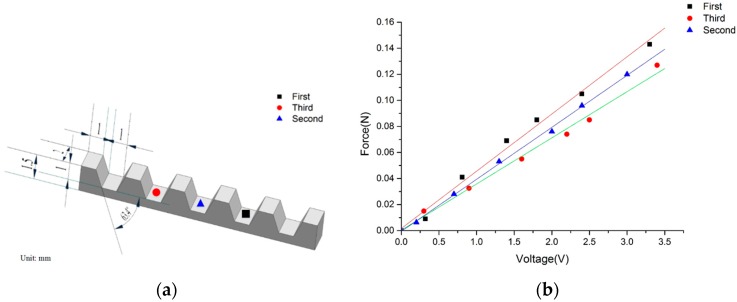
Calibration results of the microforce sensor towards different cell holding grooves (**a**) position of the force's application point; (**b**) relationship between force input and sensor output.

Figure 11 shows the relationship between force input and sensor output while the cell injection was switched between cell-holding grooves from the simply supported end to the fixed end of the PVDF beam. The slope of the force–voltage curve changes as the cell groove changes. 

The force sensitivities of the three positions are 0.0439 V/N, 0.0399 V/N and 0.0354 V/N respectively. The linearity is 1.82% full scale. As shown in Equation (9), the proportional relationship holds as the acting point of the injection force *F* varies along the beam. These results show that the sensor is consistent with the theoretical analysis and has a good linear response. The slight offset in the experimental curves might be due to the inverse piezoelectric effects of the PVDF film, which has not been considered in the model derivation.

## 4. Cell Injection Experiment on Zebrafish Embryo

### 4.1. Experiment Materials and Setup

To verify the effectiveness of the proposed microforce sensor, the PVDF sensor was adopted in the real-time injection force measurement of a robot-assisted microinjection system for manipulating zebrafish embryos. The zebrafish is one of the commonly used animal models of developmental genetics and embryonic development. Microinjection of zebrafish embryos is a standard procedure used to analyze the effects of introduced materials. The zebrafish embryos used in our cell microinjection force measurement experiments were collected in accordance with standard embryo preparation procedures [14]. The zebrafish embryo is about 800–1200 µm in diameter, with the cytoplasm and nucleus at the animal pole sitting upon a large mass of yolk. The egg coat is called the chorion, and is pierced by a needle when injecting genes into the embryo.

**Figure 12:**
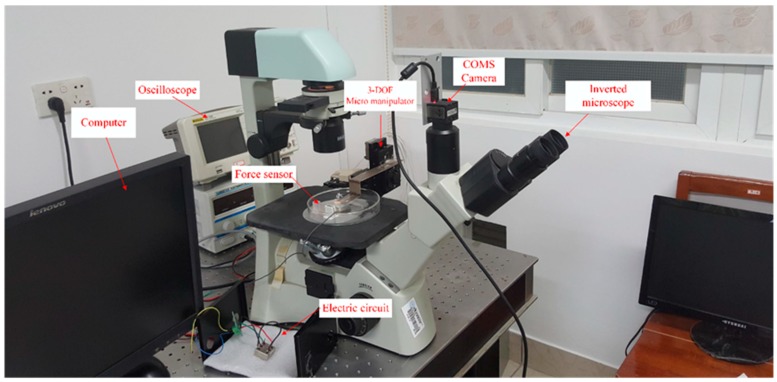
Experimental set-up for zebrafish embryo injection force measurement. 3-DOF, three degrees of freedom.

The robot-assisted microinjection system consists of the sensor, an inverted microscope (AE31, Motic, Xiamen, China) with a CCD camera (UI-1540SE-M-GL, IDS, Obersulm, Germany), and a three-degrees-of-freedom micro-robot (MP-285, Sutter, Novato, CA, USA) for controlling the needle, as shown in Figure 12. The PVDF beam creates an extrusion when the needle is injected into the embryo. The output of the PVDF charge signal is then transformed to a voltage output by the electronic circuit of Figure 6. The injection needles were fabricated by a micropipette puller (P2000, Sutter, Novato, CA, USA). The different diameters of needle tip were obtained by adjusting the laser heating time. Tip diameters of 20 µm, 40 µm, and 60 µm were selected.

### 4.2. Experiment Method and Results

We performed robot-assisted microinjection on 18 embryos. The embryos were randomly and averagely divided into three groups. For different group the diameter of the inject needle is different.

**Figure 13:**
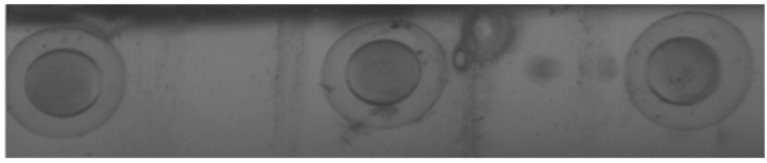
Zebrafish embryos in cell-holding device.

The first, three zebrafish embryos were placed in the trapezoidal PDMS groove, as shown in Figure 13. It can be seen that the transparency of the PDMS is satisfactory for use as the cell-holding device in a robot-assisted cell manipulation system. Compared with the horizontal bottom and top of the trapezoidal groove, the image of the groove slope is darker. This results from the surface roughness of the sloping side of the groove; it is more difficult to make the sloping sides smooth than the horizontal sections in the fabrication process.

**Figure 14:**
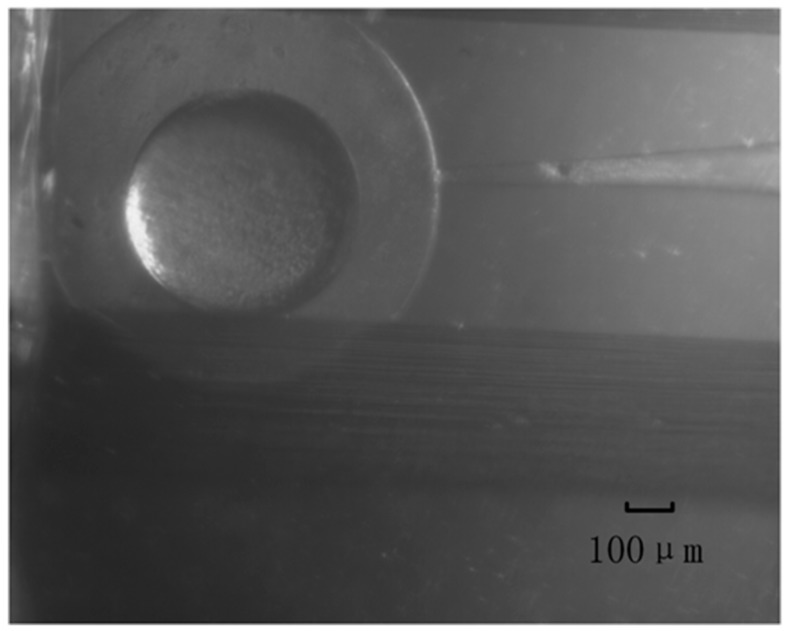
Zebrafish embryo before needle injection.

Second, the needle was moved to the 3 o’clock edge of the embryo by the manipulator. Figure 14 shows a single embryo and a needle before the robot-assisted microinjection (2× objective, 0.65× adapter). The needle microinjection speed was fixed as 2 mm/s and the move distance was 800 µm. For each group the diameter of the inject needle is 20, 40 and 80 µm, respectively. 

**Figure 15:**
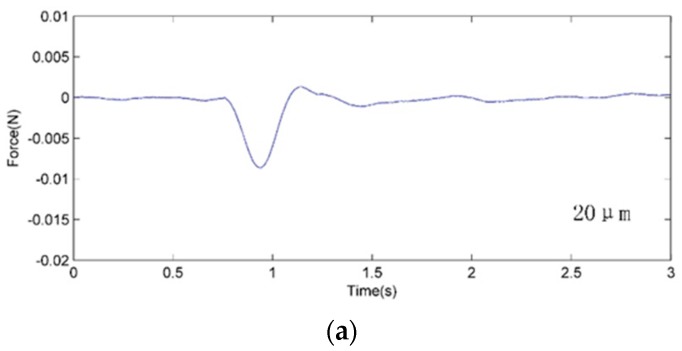

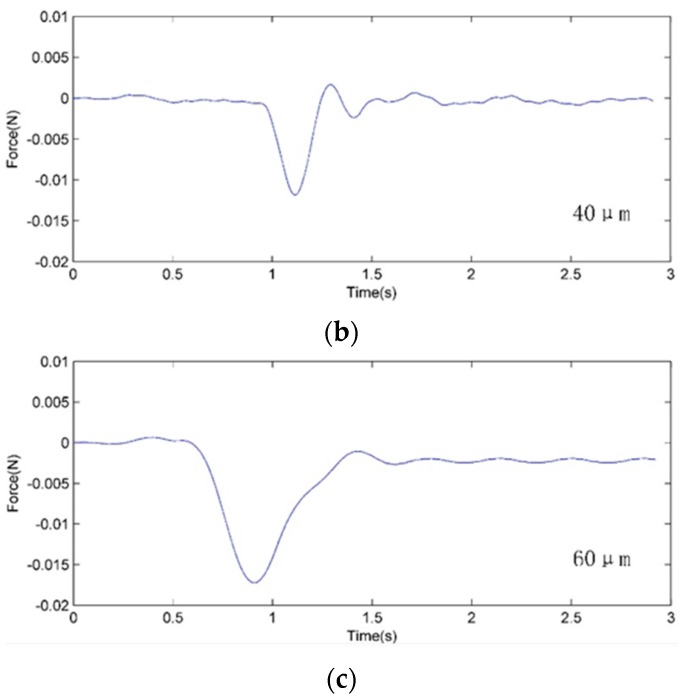
Injection force trajectories for different needle diameters. (**a**) Force curve of 20 µm diameter; (**b**) Force curve of 20 µm diameter; (**c**) Force curve of 20 µm diameter.

The typical microinjection force trajectories for different needle tip diameters are shown in Figure 15, where the vertical axis denotes the corresponding penetration force between the needle tip and embryo. It can be seen that the injection force nonlinearly increases as the needle advances. The nonlinear properties were associated with the stiffness and inner pressure of the embryo.

The force was a maximum when the embryo chorion was penetrated, which can be used as a signal for the robot to acknowledge penetration of the cell. The penetration force (peak force) increases as the needle diameter increases. The average punctuation force of are 7.3 mN, 12.1 mN and 17.9 mN corresponding to the 20, 40 and 80 µm diameter. After that, the force decreased while the chorion pressure was released. The vibration of the force trajectory during the released process is a result of the vibration of the PVDF beam.

## 5. Conclusions

To measure the real-time cell injection force trajectory in real cell microinjection experiments, a novel force-sensing beam structure was designed. Theoretical analysis showed that the force was proportional to the charge output of the piezoelectric sensor. A mechanical model of the sensor was verified experimentally. The traditional mold machining method was used to make a cell-holding device. This approach can be applied to smaller scales [15]. Finally, the effectiveness of the designed sensor when working with a micro-robotic cell manipulation system was verified experimentally.
